# Change of Exposure Response over Time and Long-Term Risk of Silicosis among a Cohort of Chinese Pottery Workers

**DOI:** 10.3390/ijerph8072923

**Published:** 2011-07-14

**Authors:** Yi Sun, Frank Bochmann, Peter Morfeld, Kurt Ulm, Yuewei Liu, Heijiao Wang, Lei Yang, Weihong Chen

**Affiliations:** 1 Institute for Occupational Safety and Health of the German Social Accident Insurance/Alte Heerstraße 111, 53757 Sankt Augustin, Germany; E-Mails: yi.sun@dguv.de (Y.S.); frank.bochmann@dguv.de (F.B.); 2 Institute of Occupational Medicine, Social Medicine and Social Hygiene, University of Cologne; Institute for Occupational Epidemiology and Risk Assessment of Evonik Industries, Kerpener Str. 62, 50937 Cologne, Germany; E-Mail: peter.morfeld@evonik.com; 3 Institute of Medical Statistics and Epidemiology, Technical University of Munich/Ismaningerstr. 22, 81675 Munich, Germany; E-Mail: kurt.ulm@tum.de; 4 School of Public Health, Tongji Medical College, Huazhong University of Science and Technology, Hongkong Road 13, Wuhan 430074, China; E-Mails: yliu@mails.tjmu.edu.cn (Y.L.); wanghaijiao2006@163.com (H.W.); leiyang@mails.tjmu.edu.cn (L.Y.)

**Keywords:** respirable silica exposure, exposure pattern, exposure-response-relationship, excess risk, regulatory risk assessment

## Abstract

An analysis was conducted on a cohort of Chinese pottery workers to estimate the exposure-response relationship between respirable crystalline silica dust exposure and the incidence of radiographically diagnosed silicosis, and to estimate the long-term risk of developing silicosis until the age of 65. The cohort comprised 3,250 employees with a median follow-up duration of around 37 years. Incident cases of silicosis were identified via silicosis registries (Chinese X-ray stage I, similar to International Labor Organisation classification scheme profusion category 1/1). Individual exposure to respirable crystalline silica dust was estimated based on over 100,000 historical dust measurements. The association between dust exposure, incidence and long-time risk of silicosis was quantified by Poisson regression analysis adjusted for age and smoking. The risk of silicosis depended not only on the cumulative respirable crystalline silica dust exposures, but also on the time-dependent respirable crystalline silica dust exposure pattern (long-term average concentration, highest annual concentration ever experienced and time since first exposure). A long-term “excess” risk of silicosis of approximately 1.5/1,000 was estimated among workers with all annual respirable crystalline silica dust concentration estimates less than 0.1 mg/m^3^, using the German measurement strategy. This study indicates the importance of proper consideration of exposure information in risk quantification in epidemiological studies.

## 1. Introduction

Silicosis, one of the earliest recognized occupational diseases, is a pathological condition of the lungs due to inhalation of particulate matter containing crystalline silica [1]. Silicosis may reflect a failure in adequate control of occupational dust exposure. Although advances in occupational safety and health make this disorder highly preventable, silicosis remains the most prevalent occupational disease worldwide [2]. The situation is particularly serious in developing countries, where millions of workers are at risk of developing silicosis. Since crystalline silica was classified as a human carcinogen by IARC in 1997 [3], a series of quantitative risk assessments has been conducted for respirable silica dust exposure, especially for low exposure levels [4–8].

Although the published exposure-response relationships and excess risk estimations have resulted in advanced risk communications, methodological limitations in published studies have stirred much debate within the scientific community. Besides the common problems in design, lack of adjustment of confounding [9], limited quality and poor comparability of exposure data between studies [10], inadequate consideration of exposure data or inappropriate biological assumptions of the exposure-response relationship in data analysis also seem to be important limitations.

Previously, the exposure-response relationship and excess risk estimations of silicosis have been based mainly on an assumption of a monotonic association between cumulative silica dust exposure and the incidence or prevalence of silicosis. The impact of exposure patterns (various combinations of long-term average respirable silica dust concentration, change of exposure concentration over time and latency) on risk estimations has, to our knowledge, never been considered. Previous analysis indicated that the lack of consideration of disease latency in data analysis may lead to a difference in the estimated health risks by as much as a factor of 10 [11]. In order to quantify the possible influence of exposure patterns on exposure-response relationships and long-term risk estimations of silicosis, an analysis was conducted on a cohort of Chinese pottery workers.

## 2. Methods

### 2.1. Design and Study Population

Details of the study design and methods, including case identification, diagnostic procedures, exposure monitoring methods, and follow-up information have been described previously [12–14]. Briefly, a cohort of silica exposed workers, established in the late 1980s, was followed for silicosis morbidity and all-cause mortality from Jan. 1, 1960 to Dec. 31, 1994 in 29 Chinese mines and factories (active employment between Jan. 1, 1960 and Dec. 31, 1974). An extended follow-up of cohort members in four pottery factories, six tungsten mines and four tin mines was conducted until Dec. 31, 2003. In this extended follow-up smoking habits were also assessed. The extended follow-up was conducted in a research cooperation between Tongji Medical College, China and the Institute for Occupational Safety and Health of German Social Accident Insurance.

The study population in this analysis are cohort members of the four pottery factories who attended the extended follow-up with the following inclusion criteria:

- Starting-date of employment after January 1, 1950 and aged over 15 years
- Minimum employment of 1 year
- Without unknown external silica dust exposure

**Figure 1:**
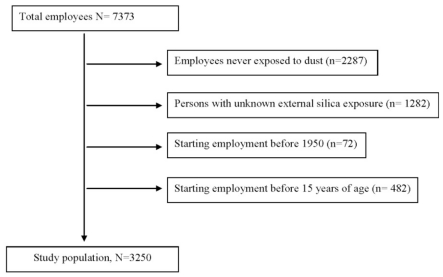
Study population.

In total, 7,373 employees joined the extended follow-up. Due to the inclusion criteria, 4,123 persons were excluded from the initial cohort. This resulted in a final cohort of 3,250 employees for analysis (Figure 1).

### 2.2. Ascertainment of Incident Cases of Silicosis

A program for detection of silicosis by regular chest radiographs was launched in China in the early 1950s. Later, this program was continued in the form of a national law in 1963 [15]. According to this law, each company was and is responsible for providing yearly chest radiographs for dust-exposed workers, and for maintaining a register for employees with silicosis. This law also makes provision for a radiographic examination of workers exposed to dust every 2–3 years even after the cessation of dust exposure [15].

Radiographic reading and diagnosis of silicosis were performed in China in each province by specially trained medical teams. Diagnostic criteria were based on a standardized radiographic grading system usually applied in China, which classifies silicosis as stage 0 (suspected cases), I, II or III. If a worker has silicosis of stage I or above, he will be defined as having definite silicosis. In an inter-reader comparison study between the Chinese and Internatinoal Labor Organisation (ILO) classification system (revised ED 1980), a good agreement of 89.3% was found between the Chinese stage I and ILO profusion category 1/1 [15].

The onset of silicosis was defined in this study as the date of the radiograph leading for the first time to classification as stage I or higher. For workers without silicosis, the end of the follow-up-period was defined as the date of the last X-ray examination.

### 2.3. Occupational Exposure Assessment

More than 100,000 historical industrial hygiene data dating back to the 1950s are available for total dust, particle size and percentage of free silica in the pottery factories studied. These data were used in this study to create a job exposure matrix (JEM) for average total dust for each calendar year. Approximately 220 facility/job title combinations over 30 calendar periods, starting in 1950, were available. Over 60 percent of facility, job title, and calendar year data were estimated based on direct monitoring data. The remainder were estimated from monitoring data for similar jobs or data for the same job at different times, with adjustment for other historical exposure information and task descriptions for the job title. A detailed description of the JEM has been published elsewhere [14].

To estimate cumulative and long-term average dust exposures, complete individual work histories for each study subject were assessed using employment records in the personnel files of the mining companies and factories involved.

For estimation of the respirable silica dust exposures, special monitoring programs were designed to compare the Chinese total dust with the US respirable crystalline silica dust concentrations based on side-by-side measurements during 1988–1989. Conversion factors between Chinese total dust and US respirable crystalline silica dust exposure have been published in the past [16]. These conversion factors were updated in an analysis by the addition of recent measurements data from 2000 to 2006. The new analysis confirmed that the contents of free crystalline silica did not change substantially over time. However, respirable dust measurement values based on the German measurement strategy are about twice those based upon the US measurement strategy [17].

Respirable crystalline silica dust exposures were estimated by multiplication of the total dust exposures by the corresponding conversion factors (respirable crystalline silica concentration = total dust concentration × conversion factor).

### 2.4. Statistical Analysis

The statistical analysis was conducted in two steps. In the first step, exposure-response relationships between respirable crystalline silica dust exposure and the incidence of silicosis were quantified by Poisson regression analysis adjusted for age at first exposure (classified in three categories in the model), sex and smoking (ever *vs.* never). Exposure patterns were considered time-dependently in the model and presented as various combinations of long-term average exposure, highest exposure ever and time since the first exposure (time since first exposure is not identical with follow-up duration, since the starting date of employment is not the starting date of follow-up). Highest exposure ever was defined as the highest annual average concentration ever experienced during the working lifetime. Since the main purpose of this analysis is the quantification of absolute risk of silicosis at low exposure levels rather than the identification of possible threshold values of silica dust exposures, no thresholds were assessed in this study [18]. Calculation of time-dependent person-years was performed with the help of Wood’s algorithm [19]. The effect estimates were calculated by PROC GENMOD (SAS 9.2) [20].

In the second step, the expected long-term risk of silicosis was quantified for a selected target population based on the estimated exposure-response relationships [21].

In this analysis, the German population was chosen as the target population. The silicosis risk was quantified based on a typical exposure situation in which workers begin employment aged 20 years and retire aged 65 years with a working life of total of 45 years. The risk of silicosis at each working year (or age) can be quantified by the Poisson regression model (depending on the exposure level, latency, smoking *etc.*). The long-term risk was estimated by cumulating the risks of silicosis over the whole working life between the ages of 20 years and 65 years, corrected for natural deaths within the target population (life table for male in Germany in 1995);

$$\begin{array}{c} R_{lifetime}=\frac{\sum_{i=20}^{65}N_{i}*{\hat{I}}_{i}}{N_{20}}\\ N_{i+1}=N_{i}*(\text{exp}(-\lambda_{i})-{\hat{I}}_{i})\\ {\hat{I}}_{i}=\text{exp}({\hat{\beta }}_{0}+{age}_{i}{\hat{\beta }}_{{age}_{i}}+\sum_{j=1}^{k}x_{j}{\hat{\beta }}_{j}) \end{array}$$

where:

R_long-term_: Estimated long-term risk of silicosis over the whole working life between the ages of 20 years and 65 years;

N_i_ : Risk set at age i;

*Î_i_*: Incidence of silicosis at age i estimated by the Poisson regression model;

λ_i_: All-cause mortality of target population at age i;

x_j_: Other parameters considered in the Poisson regression model;

*β̂_j_*: Effect estimates of variables x_j_ in the Poisson regression model.

## 3. Results

Table 1 provides a description of the study population. In total, 3,250 workers were followed for silicosis morbidity for a median duration of 36.6 years. During the follow-up period, a silicosis cumulative incidence of 15.5% were identified. In addition to dust exposure, the smoking status was assessed almost completely. About 60% of cohort members had smoked at some point.

Information on both total dust and respirable crystalline silica dust exposure is presented in Table 2. On average, respirable crystalline silica dust accounted for around 4% of total dust exposure. The long-term average respirable crystalline silica dust exposure varied between 0 (below the detection limit) and 1.16 mg/m^3^, with a highest exposure ever up to 1.95 mg/m^3^. The average duration of respirable crystalline silica dust exposure was around 28 years, with a longest duration of more than 46 years.

**Figure 2:**
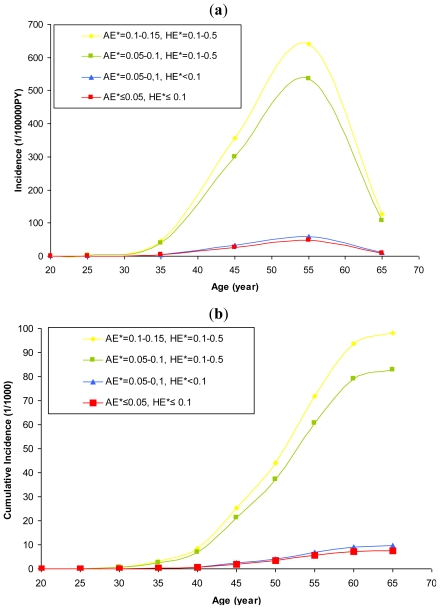
Exposure-response relationship between respirable crystalline silica dust exposure and silicosis over time (both gender, non-smoker). (**a**) Estimated incidence density of silicosis; and (**b**) Estimated cumulative incidence of silicosis. * AE = long-term average exposure, HE = highest exposure ever (mg/m^3^). Highest exposure ever was defined as the highest annual average concentration ever experienced during the working lifetime.

Tables 3 and 4 provide the results of the Poisson regression analysis for total dust and respirable crystalline silica dust exposure, respectively. The goodness-of-fit of the models was evaluated in a comparison between the observed and the estimated number of silicosis cases for each covariate in the model. They show excellent agreement (results not shown). The risk of silicosis increased consistently with increasing respirable crystalline silica dust exposure. Similar trends in exposure-response relationships can be observed for both total dust and respirable crystalline silica dust exposures. The estimated effects shown in Table 4 were used for calculation of the incidence rate of silicosis and the exposure-response relationship as shown in Figure 2.

Figure 2a shows a dynamic change in silicosis incidence among workers exposed to dust over their whole working lives between the ages of 20 years and 65 years. Where workers begin employment aged 20 years, virtually no risk of silicosis is expected during the first 10 years of their working lives. Thereafter, the risk of silicosis increases rapidly, peaking around 35 years after the onset of exposure. After this point, the incidence decreases. This figure expresses the estimated exposure-response relationship between respirable crystalline silica dust exposure and silicosis over time.

The estimated exposure-response relationship between respirable crystalline silica dust exposure and silicosis was also presented by the cumulative risk estimator as shown in Figure 2b. The cumulative risk curve shows a typical s-form. The peak of each curve represents the estimated long-term risk of silicosis.

The long-term risks of silicosis under various exposure patterns are summarized in Table 5. Under the same exposure conditions (long-term average exposure), workers having short-time higher exposure in their work history tend to have a higher risk of silicosis. In contrast, workers having constant lower exposure over a longer exposure duration have a much lower silicosis risk. A baseline risk of silicosis of 13.6/1,000 was identified in this analysis even among workers without dust exposure. Presumably, this finding indicates a false positive diagnosis in silicosis detection (e.g., due to smoking or other reasons). Given this baseline risk, an “excess” risk of silicosis of 0.9/1,000–1.9/1,000 was estimated among workers if all annual respirable crystalline silica dust concentrations are kept below 0.1 mg/m^3^ which implies that the long-term respirable crystalline silica dust exposure is limited at the same time to 0.1 mg/m^3^.

## 4. Discussion

Previous analysis in the quantification of silicosis risk has often suffered from two types of limitation: limited historical exposure monitoring data; and poor comparability of exposure information reported in various studies or countries—differences in average crystalline silica dust exposure between industrialized countries of up to a factor of 12 were reported even in the same industrial sector and calendar periods [10]. This difference is more likely a reflection of the different exposure control measurement strategies used in various countries (e.g., selection of dust sampling site, compliance *vs.* non-compliance measurements) rather than of the true differences in their related working conditions. This problem often makes comparison of studies and a valid interpretation difficult.

Compared with most silicosis studies published to date, this study has advantages, including the ascertainment of incident cases of silicosis via well-established silicosis registries, clear definition of follow-up end-point for both silicotic and non-silicotic workers (via periodical X-ray examination), long-term follow-up, complete ascertainment of smoking habit, and in particular, the large quantity of well-documented historical exposure monitoring data.

The comprehensive historical measurement data permit, for the first time, a detailed quantification of the dynamic change in exposure-response relationship between various patterns of respirable crystalline silica dust exposure and the incidence of silicosis over time. The results of this analysis indicate that the risks of silicosis depend not only on respirable crystalline silica dust exposure levels, but also on their related exposure patterns and time. “Time” was presented in this analysis by two variables: “age at first exposure” and “time since the first exposure”. Previous analysis indicates that, age as a parameter of biological time have an independent effect on silicosis appearance [21].The combination of age, exposure duration and latency allows flexibility in estimating the influence of time on the occurrence of silicosis. The peaks presented in Figure 2a demonstrate the influence of latency on the occurrence of silicosis. The decreased incidences of silicosis after the peaks exhibit a typical healthy-survivor effect (risk decreases even though the residence time of silica dust in the lung continues to increase with time).

To ensure a valid exposure assessment in this study, workers with higher exposure uncertainties (unknown external dust exposure, employment before 1950 or below the age of 15 years) were excluded from the data analysis. The conversion factors between total dust and respirable dust exposures were compared by the use of different measurement strategies. The content of crystalline silica in the dust was also quantified by comparison between different laboratories. Our analysis demonstrates that comparable crystalline silica content in the dust was found in different laboratories in the USA and Germany. However, conversion factors estimated with use of the German measurement strategy were about twice as high as those estimated with use of the US measurement strategy. In this analysis, conversion factors between total dust and respirable dust exposures were quantified on the basis of the German measurement strategy.

To quantify the stability of the effect estimates in this study, we compared the results by using the updated conversion factor (based on measurement data conducted during the time 1988–1989 and 2000–2006) with previously published conversion factors (based only on the 1988–1989 measurements). Little change was found for the estimated long-term “excess” risk with use of the different conversion factors.

One main purpose of this analysis is the quantification of long-term risks of silicosis for a target population by use of the estimated effects of respirable crystalline silica dust exposures derived from a well-conducted epidemiological study. Since the impact of silica dust exposure on the occurrence of silicosis is unlikely be influenced by the population studied. Pooled analysis based on various type of population groups have been conducted so far to estimated the long-term risk of silica related health issues [6,7]. In this analysis, we quantify the long-term risk of silicosis based on a cohort of Chinese pottery workers.

The long-term risk estimates developed in this study describe the probability of contracting silicosis due to a long-term exposure to respirable crystalline silica dust under special exposure levels or patterns. These risk estimates are accumulated over the whole working life. We applied the NIOSH approach [22], ignoring certain recommended refinements [23].

Previous estimations of the long-term risk of silicosis were based mainly on the assumption of a monotonic exposure-response relationship between cumulative crystalline silica dust exposure and the incidence or prevalence of silicosis. One important limitation of this assumption is that, time (especially latency) was not considered in the analysis. For a given cumulative exposure, exposure could be accumulated over 5 years (higher exposure) or 40 years (lower exposure). Depending on the time (especially latency) given in the data, the estimated risks may either be underestimated or overestimated. Risk quantifications based on this assumption may therefore be misleading.

In this analysis, the exposure-response relationship was presented by the incidence of silicosis (under a certain exposure pattern) over time. The long-term risk of silicosis was quantified by accumulation of the risks of the target population strictly “over time” from the ages of 20 years to 65 years, as shown in Figure 2b. Since the risk quantification is corrected for competing deaths by application of the life table of the target population, this analysis actually gives the expected long-term risk of silicosis of the target population, given the population were exposed.

Overall, a baseline risk of silicosis of 13.6/1,000 was found in this study, even among workers not subject to dust exposure. Possible reasons for this finding may partly be associated with the common diagnostic uncertainty of early detection of silicosis [24,25]. This is consistent with previous findings among workers not subject to dust exposure. These show that 0.2% to 11.7% of workers not subject to dust exposure have small lung opacities in radiographs which are likely to be diagnosed as silicosis or pneumoconiosis [26,27]. In this study, one case of silicosis was also identified in an individual not subject to dust exposure (excluded from the data analysis) and in two cases, the ‘highest annual exposure ever’ was below 0.1 mg/m^3^.

## 5. Conclusions

Compared to the baseline risk, a long-term “excess risk” of silicosis of 0.9/1,000 to 1.9/1,000 is expected among employees with a long-term respirable crystalline silica dust exposure below 0.1 mg/m^3^. This estimate is conservative, since it does not take potential threshold effects into account [17]. It is worth noting that, this estimate is quantified by using the German dust measurements strategy. Similar risk will be expected for an exposure level of 0.05 mg/m^3^ by using the US dust measurement strategy.

## Figures and Tables

**Table 1:** Description of the study population.

		Potteries
N		3,250
Age (years)	Start of follow-up: mean (SD)	25.5 (6.5)
	End of follow-up: mean (SD)	60.3 (10.6)
Sex (% female)		24.4
Follow-up-duration (years)	Mean (SD)	34.8 (8.9)
	Median (range)	36.6 (1.1–45)
Silicosis	n (%)	504 (15.5)
Smoking	Information available (%)	99.9
	Non-smoker (%)	39.6
	Ex-smoker (%)	28.6
	Smoker (%)	31.8

**Table 2:** Description of dust exposures.

	Total dust	Respirable crystalline silica dust
Long-term average exposure (mg/m^3^)		
Mean (SD)	6.1 (4.8)	0.27 (0.19)
Median (min.–max.)	4.8 (0–36.7)	0.22 (0–1.16)
Highest exposure ever* (mg/m^3^)		
Mean (SD)	23.5 (11.6)	0.73 (0.34)
Median (min.–max.)	23.8 (0–65.8)	0.74 (0–1.95)
Cumulative exposure (mg/m^3^-year)		
Mean (SD)	226.4 (171.0)	7.32 (5.22)
Median (min.–max.)	183.7 (0–861.7)	6.20 (0–26.6)
Exposure duration (years)		
Mean (SD)	27.8 (7.4)	
Median (min.–max.)	27.8 (1.1–46.4)	

* Highest exposure ever was defined as the highest annual average concentration ever experienced during the working lifetime.

**Table 3:** Results of Poisson-regression analysis based on total dust exposure.

		No. of silicosis	Person-years	β	95% CI
Intercept				−11.58	−13.56, −9.58
Age at first exposure (years)	≤20	210	49,286	0	–
	20–30	225	54,441	0.03	−0.16, 0.22
	>30	69	9,272	0.51	0.24, 0.78

Sex	female	39	29,031	0	–
	male	465	83,960	1.31	0.95, 1.66

Smoking	never	135	47,839	0	–
	ever	369	65,159	0.18	−0.04, 0.39

Highest total dust exposure ever (mg/m^3^)*	1. tertile	166	46,256	0	–
	2. tertile	150	38,424	0.01	−0.22, 0.25
	3. tertile	188	28,319	0.37	0.01, 0.64

Time since the first exposure (years)	0–9	1	21,463	0	–
	10–19	30	31,597	2.92	0.92, 4.91
	20–29	208	29,510	4.90	2.93, 6.86
	30–39	244	20,657	5.44	3.48, 7.41
	≥40	21	9,771	3.84	1.83, 5.85

Long-term average total dust exposure **	1. quintile	100	28,167	0	–
	2. quintile	100	17,902	0.37	0.09, 0.66
	3. quintile	102	19,089	0.48	0.19, 0.77
	4. quintile	101	15,132	0.66	0.34, 0.97
	5. quintile	101	32,709	0.05	−0.30, 0.40

* Highest exposure ever was defined as the highest annual average concentration ever experienced during the working lifetime.

* Highest exposure ever (mg/m^3^): 1. tertile: from 0 to <19.6 (total dust); 2. tertile: from 19.6 to <29.8 (total dust); 3. tertile: from 29.8 to 65.8 (total dust).

** Long-term average exposure (mg/m^3^): 1. quintile: from 0 to <3.94 (total dust); 2. quintile: from 3.94 to <5.90 (total dust); 3. quintile: from 5.90 to <8.67 (total dust); 4. quintile: from 8.67 to <11.73 (total dust); 5. quintile: from 11.73 to 37.7 (total dust).

**Table 4:** Results of Poisson-regression analysis based on respirable crystalline silica dust exposure.

		No. of silicosis	Person-years	β	95% CI
Intercept				−13.99	−16.41, −11.56

Age at first exposure (years)	≤20	67	61,766	0	–
	20–30	390	36,849	0.02	−0.17, 0.21
	>30	47	14,384	0.48	−0.21, 0.75

Sex	female	39	29,031	0	–
	male	465	83,960	1.40	1.04, 1.76

Smoking	Never	135	47,839	0	–
	ever	369	65,159	0.16	−0.06, 0.37

Highest silica exposure ever (mg/m^3^)*	<0.10	2	8,386	0	–
	0.1–0.5	194	45,524	2.08	0.66, 3.50
	>0.5–1.0	283	50,849	2.30	0.87, 3.73
	>1.0	25	8,237	1.27	−0.22, 2.76

Time since the first exposure (years)	0–9	1	21,463	0	–
	10–19	30	31,597	2.95	0.96, 4.94
	20–29	208	29,510	4.99	3.02, 6.95
	30–39	244	20,657	5.58	3.61, 7.55
	≥40	21	9,771	3.96	1.95, 5.97

Long-term average silica exposure (mg/m^3^)	<0.05	18	10,657	0	–
	0.05–<0.10	35	9,811	0.24	−0.33, 0.82
	0.10–<0.15	63	11,500	0.40	−0.13, 0.93
	0.15–<0.20	85	14,459	0.59	0.07, 1.11
	20–<0.30	133	21,977	0.75	0.24, 1.27
	0.30–<0.40	95	16,155	0.84	0.29, 1.38
	>=0.40	75	28,439	0.50	−0.07, 1.07
	0.05 mg/m^3^ increase			0.07	0.02, 0.11

* Highest exposure ever was defined as the highest annual average concentration ever experienced during the working lifetime.

**Table 5:** Estimated long-term risk of silicosis by respirable crystalline silica exposure patterns in the low exposure region.

Highest silica exposure ever (mg/m^3^)*	Long-term average silica exposure (mg/m^3^)				
	0	0.05	0.10	0.15	0.20
Long-term “excess” risk (compared to baseline)					
<0.1		0.9/1,000	1.9/1,000		
0.1–0.5		138/1,000	147/1,000	157/1,000	
>0.5–1.0				179/1,000	191/1,000
Long-term risk					
<0.1	13.6/1,000	14.5/1,000	15.5/1,000		
0.1–0.5		152/1,000	161/1,000	171/1,000	
>0.5–1.0				193/1,000	205/1,000

* Highest exposure ever was defined as the highest annual average concentration ever experienced during the working lifetime
